# Serum Triglyceride Levels Independently Contribute to the Estimation of Visceral Fat Amount Among Nondiabetic Obese Adults

**DOI:** 10.1097/MD.0000000000000965

**Published:** 2015-06-12

**Authors:** Chiao-Yu Huang, Hsien-Liang Huang, Kuen-Cheh Yang, Long-Teng Lee, Wei-Shiung Yang, Kuo-Chin Huang, Fen-Yu Tseng

**Affiliations:** From the Department of Family Medicine (C-YH, L-TL, K-CH), National Taiwan University Hospital, Taipei; School of Medicine (H-LH), Fu Jen Catholic University, New Taipei City; Department of Family Medicine (K-CY), National Taiwan University Hospital Hsin-Chu Branch, Hsin Chu City; Department of Internal Medicine (W-SY, F-YT), National Taiwan University Hospital; Graduate Institute of Clinical Medicine (W-SY), College of Medicine, National Taiwan University, Taipei; and Graduate Institute of Clinical Medical Science (K-CH), China Medical University, Taichung, Taiwan.

## Abstract

Determining the visceral fat amount is important in the risk stratification for the prevention of type 2 diabetes and obesity-related disorders. The area-based measurement of visceral fat area (VFA) via magnetic resonance imaging (MRI) is an accurate but expensive and time-consuming method for estimating visceral fat amount. The aim of our study was to identify a practical predictive parameter for visceral obesity in clinical settings.

In this cross-sectional study, we recruited 51 nondiabetic obese (body mass index [BMI] ≥ 27 kg/m^2^) adults in Taiwan (21 men and 30 women, mean age 35.6 ± 9.2 years, mean BMI 33.3 ± 3.9 kg/m^2^). VFA was quantified by a single-slice MRI image. Anthropometric indices and biochemical parameters including fasting plasma glucose, serum level of alanine aminotransferase, and lipid profiles were measured. The associations between different variables and VFA were analyzed by linear regression analysis.

Increases in BMI, waist circumference, serum levels of alanine aminotransferase and triglycerides (TGs), and decreased serum levels of high-density lipoprotein cholesterol were correlated with larger VFA. After adjustment for age, sex, and anthropometric indices, only serum TG level remained as an independent correlate of VFA. Besides demographic and anthropometric indices, adding TG level may explain a greater variance of VFA. In stepwise multivariate regression analysis, male sex, age, waist circumference, and serum TG level remained significant predictors of VFA. In a subgroup analysis among subjects with BMI ≥30 kg/m^2^, similar results were demonstrated and serum TG level remained as significant independent correlates of VFA in all of the predictive models.

Among nondiabetic obese adults, serum TG level was positively associated with VFA. The combination of sex, age, anthropometric indices, and serum TG level may be used to estimate VFA in clinical settings.

## INTRODUCTION

Overweight and obesity are the fifth leading risk for global deaths.^1^ Obesity may increase diabetes, ischemic heart disease, and certain cancer burdens.^1^ Clinically, obesity is remarkably heterogeneous,^2^ and different fat distribution patterns determine different levels of obesity-related risks.^3^ Excess visceral adipose tissue (VAT) accumulation is correlated with an increased risk of developing type 2 diabetes and cardiovascular diseases.^4,5^ People with visceral obesity usually had alteration in serum biochemical parameters, such as impaired fasting glucose, abnormal liver function, and dyslipidemia.^4,6–11^ Determining the degree of VAT accumulation is important in the diabetic or cardiovascular disease risk stratification.

Visceral fat area (VFA) measured by magnetic resonance imaging (MRI) is an accurate, radiation-free method for the quantification of visceral fat amount.^12–14^ Larger VFAs are associated with increased VAT accumulation and higher risks of obesity-related disorders.^15,16^ Measurement at the L4–L5 intervertebral disk site has been commonly accepted previously, whereas recent studies suggested a single-slice image at L3, or 5–10 cm above L4–L5, could be a better proxy for total VAT volume.^14–18^ However, increased cost and time render this technique impractical in clinical settings and in epidemiological studies.

In general population, simple anthropometric indices, such as body mass index (BMI) and waist circumference (WC), have been proposed as surrogate markers to estimate visceral adiposity.^19–21^ However, a study by Lemieux et al^19^ demonstrated that the threshold value of WC corresponding to excess visceral fat may be different by age, sex, and the degree of obesity, and few studies have investigated the validity of these alterative measurements among obese subjects. Moreover, there is a lack of a sufficient amount of data reporting the prediction of VAT accumulation by a combination of anthropometric indices and serum biochemical markers. The present study investigates the relationships between VFA and different variables, including anthropometric indices and serum biochemical parameters, among nondiabetic obese subjects. We aimed to identify an inexpensive and practical method for estimating visceral fat amount among nondiabetic obese populations in clinical settings.

## SUBJECTS AND METHODS

### Study Subjects

We recruited obese adults from patients in the weight-control clinics at the National Taiwan University Hospital in 2009. Obesity has been defined by the World Health Organization (WHO) as BMI ≥30 kg/m^2^. However, the WHO expert consultation concluded that the proportion of Asian people with a high risk of diabetes and cardiovascular disease is substantial at BMIs lower than the existing WHO cut off point.^22^ Although available data do not necessarily indicate a clear BMI cut off point for all Asians for obesity, the cut off point for high risk^22,23^ varies from 26 to 31 kg/m^2^. Accordingly, the Department of Health in Taiwan defined obesity as BMI ≥27 kg/m^2^ for Taiwanese adults.^24^ In this study, we recruited individuals aged ≥20 years and obese with their BMI ≥27 kg/m^2^. Complete clinical history was recorded, including current medical condition and medical history (comorbidities, surgical history, and any major cardiovascular events), use of medications, and alcohol consumption. We used the clinical records as the sources and methods for the selection of participants and excluded subjects with diabetes or any medical condition influencing degree of obesity.^25–30^ The exclusion criteria were as follows: pregnancy; previous diagnosis of diabetes mellitus; fasting plasma glucose ≥7 mmol/L; previous history of chronic liver diseases; thyroid disease; Cushing disease; chronic viral, bacterial, or parasitic infection; severe debilitating disease or malignancy; history of surgical or medical treatment for obesity; taking medications affecting glucose, liver function, and lipid levels; and alcoholism or drug abuse. In addition, subjects whose body weight had changed by >5% within 3 months were also excluded. Fifty-one subjects aged 20 to 56 years who met the criteria were included in the study. The institutional review board at the National Taiwan University approved this study, and all subjects provided informed consent.

### Anthropometric Indices and Biochemical Analyses

Trained nurses measured height, WC (measured to the nearest 0.1 cm), and weight (measured to the nearest 0.1 kg) at the baseline visits. WC was measured midway between the inferior margin of the last rib and the crest of the ileum in a horizontal plane at standing position. All anthropometric measurements were performed twice, and the mean value was used for analysis. BMI was calculated as weight (kg) divided by height squared (m^2^) and rounded to the nearest 10th.

A venous blood sample was collected after an overnight fasting (≥8 hours from last meal) for measuring the biochemical parameters. We measured plasma glucose level and the serum levels of alanine aminotransferase (ALT), triglycerides (TGs), total cholesterol, low-density lipoprotein cholesterol (LDL-C), and high-density lipoprotein cholesterol (HDL-C). These assays were performed on a Hitachi 7250 analyzer (Hitachi, Tokyo, Japan) according to the manufacturer's instructions. Non-high-density lipoprotein cholesterol (non-HDL-C) are calculated as total cholesterol minus HDL-C, which includes TG and additional atherogenic lipoprotein moieties.^31^

### Area-Based Adipose Tissue Measurement by MRI

Subjects underwent abdominal MRI imaging at the National Taiwan University Hospital. A single-slice T1-weighted MRI scan with subjects in the supine position was performed using a 1.5-T system (Signa HDxt; GE Medical Systems, Milwaukee, WI). Image location was defined at a level of L3 (6 cm above the L4–L5 intervertebral space), which is considered one of the best single-slice measurement sites for estimating total VAT volume according to previous studies.^13,17^ Imaging parameters included a spin-echo sequence with an 11 ms echo time, a 40 cm field of view, and a 256 × 173 matrix. The slice thickness was 8 mm with a gap of 2 mm. After image acquisition, the quantification of VFA was performed by 2 experienced technicians using open source 3-dimensional slicer image analysis software version 3.2 (Surgical Planning Laboratory, Brigham and Women's Hospital, Boston, MA). The mean value of VFA by 2 technicians was used for analysis. To assess the consistency of 2 technicians measuring the same quantity, we did the reliability analysis of 2-way mixed average measures to obtain the intraclass correlation coefficient.

### Statistical Analysis

The data were summarized as frequencies or percentages (%) for categorical variables (sex) and as means, standard deviations, or ranges for continuous variables (age, anthropometric and biochemical parameters, and VFA) for analysis. Sex difference in VFA was tested for significance using unpaired *t* tests. We performed linear regression analyses with VFA as dependent variable and each anthropometric and biochemical parameters as independent variables. We used univariate models and multivariate models to investigate the association between VFA and different variables. Bidirectional stepwise regression analysis were performed using *P* value-to-remove of 0.1 and *P* value-to-enter of 0.05 to assess which variable was most useful in the estimation of VFA. Additionally, we further performed a subgroup analysis among subjects with BMI ≥30 kg/m^2^ (based on WHO definition of obesity). In order to validate our multiple linear regression models, the linearity and homoscedasticity of the residuals were confirmed using scatter plots of the residuals versus predicted values of the dependent and independent variables. The normality of the error distribution was confirmed using the Kolmogorov–Smirnov test. Any potential multicollinearity between the explanatory variables was tested by calculating the variance inflation factor (VIF).

The sample size calculation in our study assumed a maximum of 11 eligible explanatory variables in multivariate linear regression analysis with an anticipated *R*^2^ of 0.5 (50% of the variance in VFA explained by the model). Assuming an α of 0.05 with 90% power, ≥33 subjects were needed. The power analysis was performed by G∗Power version 3.1 (Faul, Erdfelder, Lang, and Buchner, Mannheim, Germany, 2007). The statistical package SPSS version 11.0 (SPSS Inc, Chicago, IL) was used for analyses. A *P* value <0.05 was considered significant.

## RESULTS

A total of 51 obese patients (BMI ≥27 kg/m^2^) with a mean age of 35.6 ± 9.2 years were enrolled in the study. Their baseline characteristics are summarized in Table 1. Among them, a subgroup of 44 subjects (19 men and 25 women) had BMI ≥30 kg/m^2^. All male subjects in our study had a WC >90 cm, and all female subjects had a WC >80 cm. The measurements of VFA by 2 technicians were highly consistent with the intraclass correlation coefficient as 0.991 (95% confidence interval 0.985–0.996, *P* < 0.0001). The mean VFA among total subjects was 255.42 ± 116.99 cm^2^. Total male subjects’ mean VFA was 335.3 ± 102.5 cm^2^, and total female subjects’ mean VFA was 198.4 ± 91.30 cm^2^. Male subjects had a significantly larger VFA (*P* < 0.001) than female subjects.

**TABLE 1:**
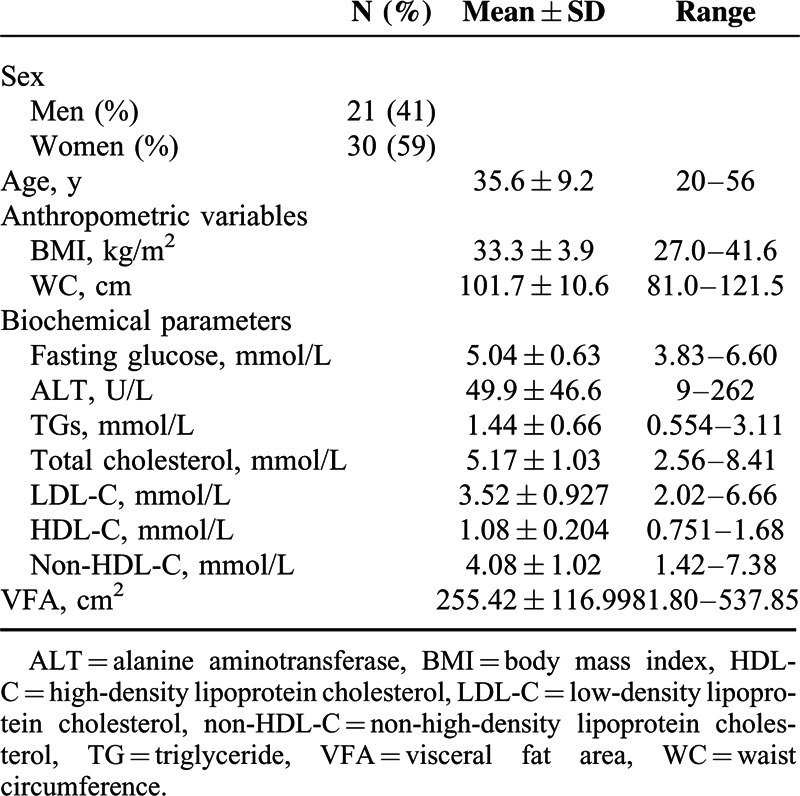
Baseline Characteristics of Study Subjects

The results of linear regression analysis are shown in Table 2. Among total subjects in univariate regression models, increases in BMI, WC, and levels of ALT and TGs and decreases in HDL-C level were correlated with increases in VFA (*P* < 0.05). Levels of LDL-C and non-HDL-C only showed the trend of positive correlation with increase in VFA without statistical significance. (*P* = 0.078 and *P* = 0.069, respectively). BMI, WC, and TG level remained correlated with VFA (*P*<0.05) after being adjusted for age and sex, whereas all the other biochemical parameters were not significantly correlated with VFA after adjustment. Independent of sex and age, the combination of BMI and WC could not explain a significantly greater variance in VFA than did either BMI or WC alone. After adjusted for age, sex, BMI, and WC, TG level remained a significant correlate of VFA (*P* = 0.007). Adding variables of serum TG level to the model of sex, age, BMI, and WC can explain an additional 13% of the variance in VFA (adjusted *R*^2^ 0.573 vs 0.466, *P* < 0.05). In the multivariate model including all independent variables, serum TG level still remained a significant correlate of VFA. The subgroup analysis among subjects with BMI ≥30 kg/m^2^ showed similar results (Table 2). Serum TG level remained significant independent correlates of VFA after adjustment in each model. None of the models exhibited any colinearity problems (VIF < 10).

**TABLE 2:**
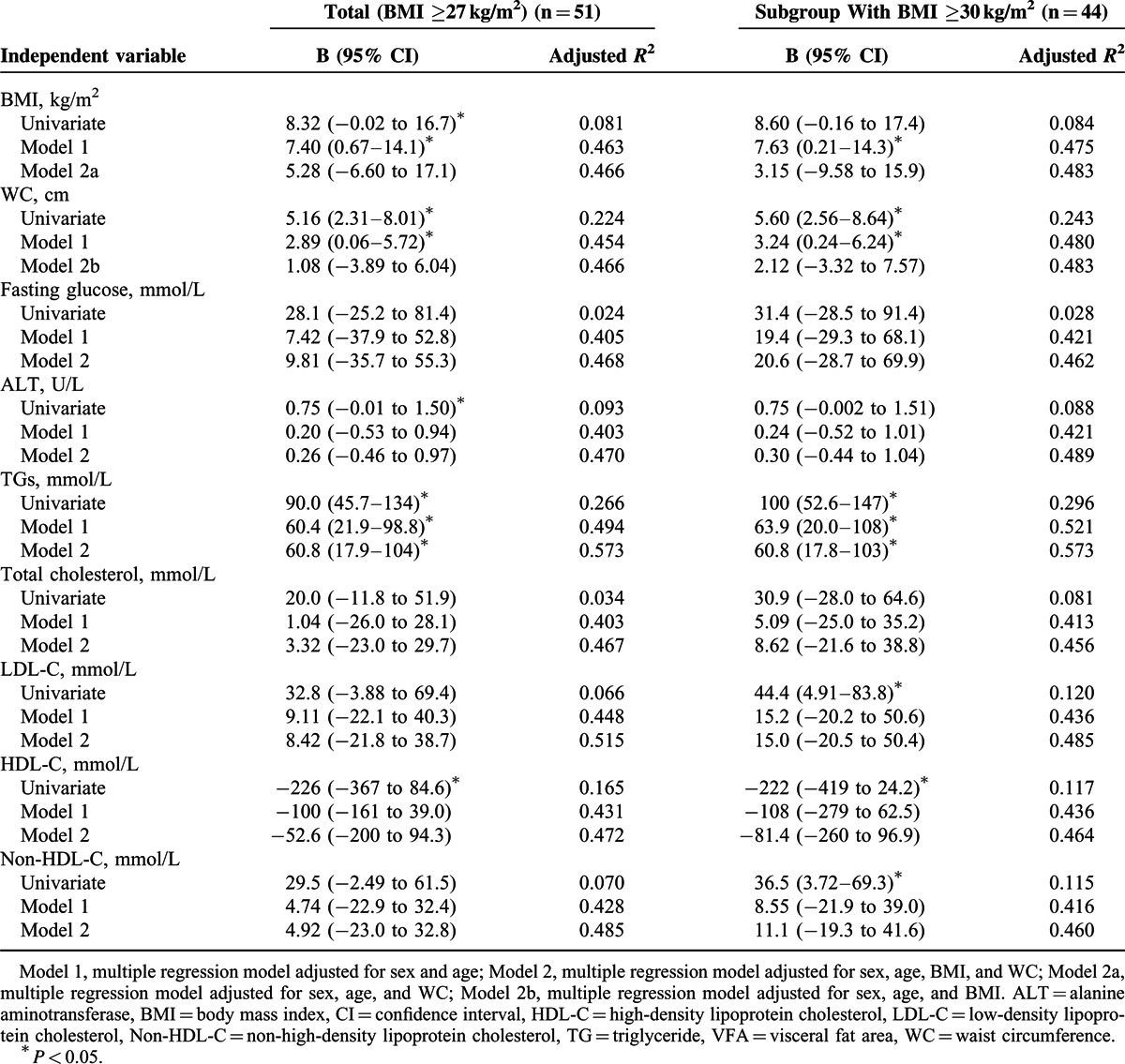
Linear Regression Analysis With VFA as Dependent Variable and Anthropometric and Biochemical Parameters as Independent Variables

The results of stepwise multiple linear regression analysis were shown in Table 3. Age, WC, and serum levels of TG of male subjects were independent correlates of VFA (*P* < 0.05), and these results are consistent both in total and subgroup subjects with BMI ≥30 kg/m^2^. Among total subjects, the regression model could explain 57.2% of variance in VFA. Among subgroup subjects with BMI ≥30 kg/m^2^, the regression model could explain 58.1% of variance in VFA. To test the linearity of age adjustment, we added age-squared term to the model. Neither coefficient of age nor age-squared term is statistically significant, and the significant correlation of VFA with WC and TGs remained unchanged.

**TABLE 3:**
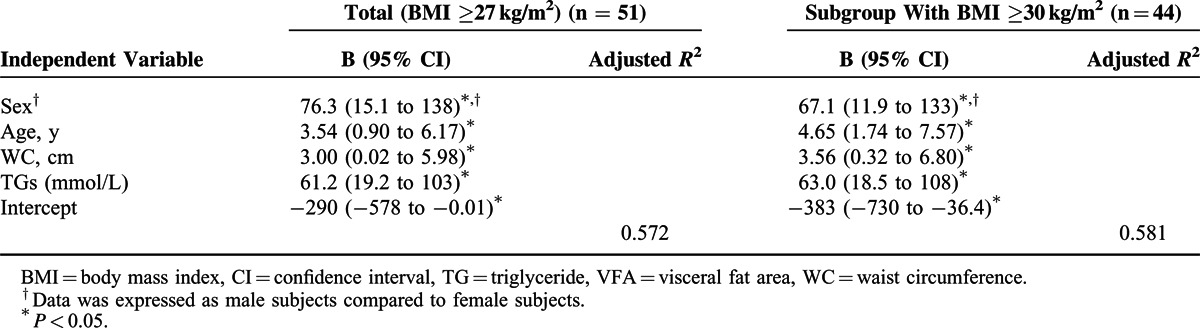
Stepwise Multiple Linear Regression Analysis With VFA as Dependent Variable and Sex, Age, Anthropometric, and All Eligible Biochemical Parameters as Independent Variable

## DISCUSSION

In the last decades, a parallel increase in diabetes was observed with the increasing incidence of obesity in Taiwan. According to the Nutrition and Health Survey in Taiwan, the prevalence of diabetes escalated from 6.2% in 1993–1996 to 7.8% in 2005–2008.^32^ The prevalence of obesity (BMI ≥ 27) also boosted from 10.1% in men and 12.7% in women in 1993–1996 to 18.9% in men and 17.1% in women in 2005–2008.^33^ In nondiabetic obese individuals, determining the visceral fat amount is important in risk stratification for diabetes. The current study identified that serum TG level along with WC may serve as dichotomous risk factors among nondiabetic obese adults in Taiwan.

It is well known that the amount of visceral fat increases with age in both sexes.^4,34^ Men are more likely to accumulate abdominal visceral adiposity then women.^4,34^ The sex- and age-related difference in visceral adiposity may explain different cardiometabolic risk profiles.^35,36^ Consistent with these reports, our analysis revealed that male sex and increases in age were independently associated with increased VFA. Our study did not indicate a postmenopausal change of visceral adiposity among women because most of the recruited female subjects were <50 years of age. Our observation highlights the importance of risk awareness in male or older nondiabetic obese subjects.

The use of anthropometric indices, such as BMI and WC, to estimate visceral adiposity has been proposed in previous studies.^19–21^ One study demonstrated that BMI and WC independently contribute to the prediction of visceral fat in the general white population and the combination of BMI and WC predict more precisely than did either BMI or WC alone.^20^ However, other studies reported the limitation of BMI in estimating visceral obesity.^2,37^ Controversy also exists regarding the predictive value of WC for high-risk abdominal obesity.^2,20,21,38^ Previous studies revealed that the correlation between WC and visceral adiposity were less significant among overweight-to-obese subgroups.^21^ More studies have suggested that WC is a reasonably good correlate of the amount of total abdominal fat, but it cannot distinguish VAT from subcutaneous adipose tissues.^2,39^ Among our obese subjects, both BMI and WC were positively correlated with VFA independent of age and sex. The combination of BMI and WC did not explain greater variance of VFA than BMI or WC did alone. In stepwise regression analysis, WC, but not BMI, remained as an important independent determinant of VFA.

Patients with increased visceral obesity usually had dyslipidemic status such as high levels of TGs, low levels of HDL-C, relatively normal total cholesterol levels, and increased non-HDL-C.^4^ The relationship between visceral adiposity and serum TG level among diabetic and nondiabetic populations has been established for several years.^7^ The association between TGs and VAT accumulation may be caused by a combination of increased TG production and impaired clearance of TGs from the circulation in visceral obesity.^4,40,41^ The hyperlipolytic state in visceral obesity may cause increasing influx of fatty acid into liver and result in the overproduction of TGs.^42^ Relatively decreased amount of subcutaneous adipose tissue may retard the clearance of dietary TGs.^2^ Similar to previous results, our study demonstrated that serum TGs level independently and significantly contributed to the estimation of VFA. Our analysis further revealed that adding serum TG level may more precisely estimate VAT accumulation than anthropometric indices alone. In consistent with a previous study including both obese and nonobese subjects,^11^ LDL and non-HDL-C only showed a trend of association with VFA without statistical significance in our study. The negative association between serum HDL-C level and VFA also became insignificant after adjustment for sex and age. All these findings emphasized the importance of serum TG levels in the estimation of VAT accumulations among an obese population.

There is increasing evidence supporting that the combination of enlarged WC and elevated serum TG levels might serve as a dichotomous risk factor.^43–49^ Similar to our study, Sam et al^44^ found that hypertriglyceridemic waist phenotype predicts increased visceral fat in diabetic subjects. In 2005, Kahn^43^ proposed “lipid accumulation product (LAP),” a simple indicator of combining WC and TGs to reflect lipid overaccumulation, especially VAT depot (LAP = [WC cm−65] × TG [mmol/L] for men and [WC cm−58] × TG [mmol/L] for women). Roriz et al^49^ demonstrated that LAP is an accurate indicator in visceral obesity discrimination among the general adult population. Many previous studies have demonstrated the predictive value of both “lipid accumulation product” and the hypertriglyceridemic waist phenotype in diabetes, cardiovascular diseases, and other cardiometabolic risks.^45–48^ In this study, when TGs and WC were replaced with the calculated LAP of our subjects in the estimation model, the calculated LAP can explain comparable variance in VFA irrespective of age and sex (adjusted *R*^2^ 0.564, *P* < 0.001). These findings confirmed the important roles of serum TG levels along with WC in estimation of visceral fat amount and in cardiometabolic risk stratification among nondiabetic obese subjects. Further study among the general population or different ethnic groups will be needed to illustrate the detailed relationships of WC and TG levels with VAT in the future.

It is known that visceral adiposity was closely related to impaired plasma glucose homeostasis and with evidence of insulin resistance.^4^ However, the association between fasting plasma glucose levels and visceral adiposity is questionable. Smith et al^9^ reported that fasting glucose levels increased with visceral adiposity among a nondiabetic general population, whereas other investigators demonstrated that the association between fasting plasma glucose and VFA was insignificant among nondiabetic obese subjects.^50^ Our study revealed that fasting plasma glucose levels were not correlated with VFA. With the aim of identifying simple markers in general medical clinics, we did not assess individual insulin sensitivity by direct dynamic euglycemic clamps or by indirect calculation from plasma insulin level in this study.

Previous studies reported the association between levels of ALT and VAT accumulation.^10,51^ Verrijken et al^51^ demonstrated that among overweight and obese patients, ALT is associated with visceral fat mass. After correction for BMI, ALT still is significantly higher in patients with increased VAT. Our study revealed that ALT was positively correlated with VFA, but the association between ALT and VFA was insignificant after adjusting for age, sex, and anthropometric indices. Our observation suggested the limited role of serum ALT level in predicting visceral fat amount among nondiabetic obese population.

There were some limitations to the present study. First, with the aims to identify a practical method to estimate VAT accumulation in clinical settings, we included known possible cardiometabolic risk-related biochemical parameters in our analysis. Whether other novel metabolic-related parameters can increase the predictability or not remains to be studied further. Second, racial differences in fat distribution have been demonstrated in a previous study.^39^ Our study subjects were nondiabetic obese Taiwanese; the generalizability of this study might be limited. Third, as a cross-sectional study, we cannot demonstrate a causal relationship by our analysis. Further studies are needed to investigate and decipher the mechanism that underlies these relationships. Finally, our relative small sample size might also limit the ability to detect significant associations between VFA and anthropometric or biochemical parameters.

## CONCLUSION

In summary, this study demonstrated the positive association between serum TGs level and visceral fat amount among nondiabetic obese subjects. Our study reinforced the importance of using the combination of demographic parameters, anthropometric indices, and serum TG level to identify obese individuals with excess visceral fat in clinical settings. This alternative method of estimating visceral fat amount may help in the prevention and management of obesity-related health risks.
